# Challenging case of severe acute asthma in a mechanically ventilated patient managed with sevoflurane

**DOI:** 10.1002/ccr3.6571

**Published:** 2023-02-23

**Authors:** Satheesh Munusamy, Seyedeh Saba Nabavi Monfared, Phool Iqbal, Ahmed Lutfe Mohamad Abdussalam

**Affiliations:** ^1^ Intensive Care Department Hamad Medical Corporation Doha Qatar; ^2^ Emergency and Trauma Pharmacy Department Hamad Medical Corporation, Qatar University Doha Qatar; ^3^ Internal Medicine Department Metropolitan Hospital Center New York New York USA; ^4^ Hamad General Hospital Doha Qatar; ^5^ Weill Cornell Medicine Qatar Qatar

**Keywords:** asthma, bronchodilators, corticosteroids, inhalation anesthesia, sevoflurane

## Abstract

Acute severe bronchial asthma is a chronic inflammatory disease characterized by hyperresponsiveness of the airways leading to bronchoconstriction. We present a case of refractory life‐threatening bronchial asthma that was managed with sevoflurane gas along with the standard treatment and achieved stability and clinical improvement through its bronchodilator and anesthetic effect.

## 1 INTRODUCTION

Acute severe asthma is a life‐threatening emergency characterized by severe tachypnea, tachycardia, and type 1 respiratory failure.^1^ According to the international standard guidelines, it is managed with bronchodilators, systemic steroids, and magnesium sulfate in emergency cases.^2^ Here, we describe a case of a 38 years old male who presented with a severe asthmatic attack resistant to conventional therapy of bronchodilators and corticosteroids. Subsequently, the patient was started on noninvasive ventilator support; he could not tolerate it and was intubated. Later the patient had a good recovery after managing with an anesthetic inhalation agent sevoflurane and mechanical ventilator support by using an anesthesia machine. This case report highlights the potential management of severe acute asthma in the critical care unit with sevoflurane.

## 2 CASE PRESENTATION

**FIGURE 1:**
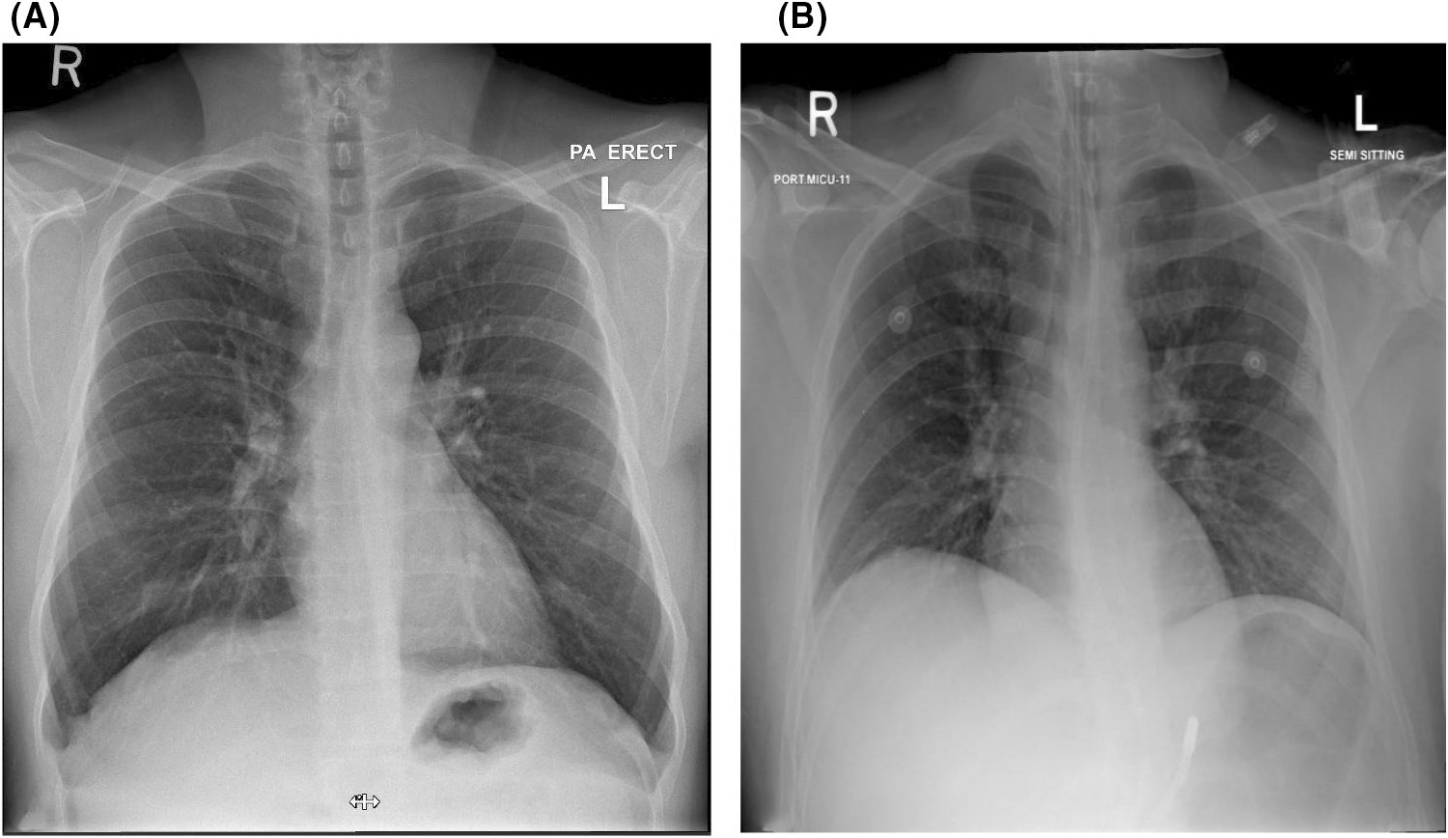
(A) Before intubation and (B) after intubation

**FIGURE 2:**
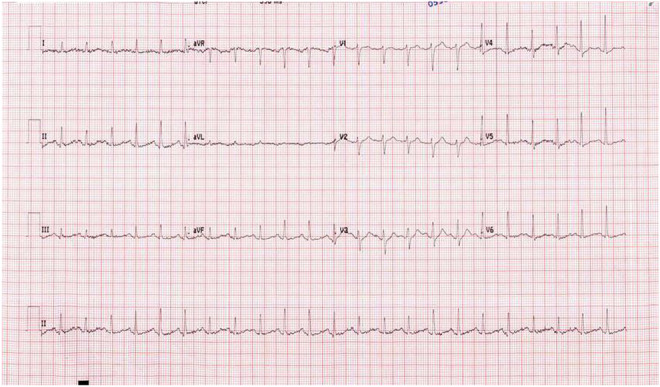
Unremarkable ECG

A 38‐year‐old man with a background of bronchial asthma presented to the Emergency Department with a 2‐day history of progressive shortness of breath following cold exposure. He was on a regular metered‐dose inhaler of Salbutamol 100 μg, one puff Q6hrly at home. He mentioned having a mild cough and audible wheezes before admission but no associated fever, rhinorrhea, cough, or upper respiratory tract symptoms. He was compliant with his medications. The patient had a recent admission with a severe asthma attack treated with intravenous corticosteroids with Hydrocortisone 200 mg three times daily, nebulizer therapy Budesonide 500 μg BID, Salbutamol 2.5 mg Q8hrly, Ipratropium bromide 125 μg QID 2 days before this admission. He was discharged home with a salbutamol inhaler, one puff Q6H, and a Budesonide inhaler 500 μg two puff BID. On day two of discharge, the patient was readmitted with complaints of cough and audible wheezes. On clinical assessment, he was oriented but was unable to complete entire sentences. He was using accessory muscles for breathing. Vitally, he had tachypnea of 35 breaths/min, oxygen saturation of 99% on 10 L of the nonrebreather oxygen mask, tachycardia of 145 beats/min, and blood pressure of 145/91 mmHg, and an oral temperature of 37.3°C. His chest XR is shown in Figure 1A and was unremarkable for any pulmonary infiltrates, pneumothorax, pleural effusion, or pathology. Initial ECG was also unremarkable, as mentioned in Figure 2. Initial arterial blood gas revealed respiratory acidosis, pH 7.314, PCO_2_ 72 mmHg, PO_2_ 132 mmHg and the bicarbonate level 32 mEq/L. Salbutamol 2.5 mg Q2hrly and Ipratropium bromide 125 μg Q2hrly nebs were given immediately.

Nevertheless, the patient kept deteriorating clinically. He was given 4 g of magnesium sulfate along with a trial of noninvasive ventilation (NIV) with the settings of ST (spontaneous timed) mode, PS (pressure support) 12, PEEP (positive end‐expiratory pressure) 6, and FiO_2_ 40%. Arterial blood gases showed worsening respiratory acidosis—pH 7.130, PCO_2_ 83 mmHg, PO_2_ 111 mmHg, and HCO_3_ 30 mEq/L. Due to the increased work of breathing and drowsy state of the patient, he was intubated and started on mechanical ventilation with volume‐controlled mode, and settings of tidal volume 450 ml, respiratory rate of 14 breaths/min, PEEP of 5 cm·H_2_O, and FiO_2_ of 60%. Chest XR postintubation is shown in Figure 1B. The flow volume scale was not touching the baseline due to severe bronchial constriction. The metered‐dose inhaler was started with bronchodilator therapy and steroids (Salbutamol 2.5 mg Q4hrly through the endotracheal tube). Auto‐PEEP showed 4 cm·H_2_O. Settings were altered as Inspiratory (I): Expiratory (E) ratio from 1:2.5 to 1:5. Secretions were cleared with closed inline suctioning regularly. Two days later, the patient did not show any improvement. Subsequently, it was decided by the ICU team to give bronchodilator therapy through an anesthetist agent, sevoflurane, in‐between 0.5% and 2% for 48 h. After starting inhaling sevoflurane patient, the peak pressure started to come down. Presevoflurane therapy and postsevoflurane therapy pressures are summarized in Table 1. The arterial blood gas showed an improvement in ventilation. The comparison of ABG values is also mentioned in Table 2. The patient gradually improved and was eventually extubated without any complications.

**TABLE 1:** The comparison of ventilator status from pretherapy and post‐therapy treatment with sevoflurane

Pressures	Pretherapy	Post‐therapy
Ppeak	48 cm·H_2_O	30 cm·H_2_O
Pplat	22 cm·H_2_O	23 cm·H_2_O
Stat. comp	21.6 cm·H_2_O	26 cm·H_2_O
Auto‐PEEP	4 cm·H_2_O	0

Abbreviations: Ppeak, peak pressure; Pplat, plateau pressure; Stat. comp, static lung compliance.

**TABLE 2:** The ABG values from pretherapy and postsevoflurane therapy treatment

ABG	Pretherapy	Post‐therapy
pH	7.314	7.443
PCO_2_	72 mmHg	38 mmHg
PO_2_	132 mmHg	102 mmHg
HCO_3_	32 mEq/L	25.9 mEq/L
Lactate	0.40	0.40

Abbreviations: HCO_3_, bicarbonate; PCO_2_, partial pressure of carbon dioxide; pH, hydrogen ion; PO_2,_ partial pressure of oxygen.

## 3 DISCUSSION

Severe bronchial asthma can be life‐threatening if not timely managed. However, patient response varies even on a standard guideline treatment. Ng et al.^3^ described acute asthma as a dangerous disease‐causing acute dyspnea leading to increase work of breathing. In the United States, 1.8‐million asthma attack cases visit the emergency department annually. Nearly 10 cases of death occur per day.^4^ Most patients will recover with standard treatment. However, few cases reported resistant status‐asthmaticus, which responded only to sevoflurane and the standard treatment.^3^ Our case describes the successful treatment of inhaled anesthesia gas in addition to standard treatment. Inhaled anesthesia gas (sevoflurane) has been shown to reduce Auto‐PEEP, bronchial constriction, and dynamic hyperinflation.^4^ sevoflurane has a rapid bronchodilator effect and smooth muscle relaxant effect, easily ventilating the patient without asynchrony. sevoflurane is usually used in anesthesia to sedate the patient during a procedure; in selected cases, only using sevoflurane in critical care settings.^5^ The main drawbacks of sevoflurane use are its high cost, monitoring, and scavenging of exhaled gas. With conservative anesthesia devices, the exhaled gas and sevoflurane can be filtered and reused.^2^ Intravenous sedation could be reduced or discontinued during sevoflurane administration through ventilator support.^6^ Soukup et al. evaluated the impact of sevoflurane during long‐term treatment of critical care unit patients, which showed high effectiveness on patient safety and reduced weaning time in comparison with standard conventional intravenous sedation concept.^7^ Routine use of sevoflurane is easily feasible, effective, and safe, has a short awakening time, and has an adequate bronchodilator effect.^8^ Current studies show that sevoflurane has significant bronchodilator properties and is an effective treatment option for severe acute asthma before rescue therapies.^9^ The beneficial effect of sevoflurane in our case is supported by alveolar unit and distal airway dilation, which reduce distortion of the surrounding parenchyma and amount of alveolar collapse and finally reduce viscoelastic stress adaptation. sevoflurane has a rapid bronchodilator effect and muscle relaxant properties by reducing intracellular calcium in smooth muscle cells to ventilate the patient efficiently without asynchrony.^10^ Despite the presence of nephrotoxic metabolites upon sevoflurane degradation, it can be considered a safe option in long‐term use and challenging intubation case scenarios.^11^, ^12^ This case aims to show there is an effect of sevoflurane for acute severe asthma cases treatment in addition to standard treatment.

## 4 CONCLUSION

Our objective is to highlight the potential of sevoflurane in the management of severe acute asthma refractory to standard management in mechanically ventilated patients. sevoflurane can be considered a lifesaving add‐on therapy to the standard treatment of severe asthma in resourceful countries. Considering endotracheal intubation should not be delayed in severe asthma to avoid fatal outcomes. Further meta‐analysis and studies are recommended to evaluate the long‐term efficacy and clinical applicability.

## AUTHOR CONTRIBUTIONS

**Satheesh Munusamy:** Conceptualization; investigation; methodology; writing – original draft; writing – review and editing. **Seyedeh Saba Nabavi Monfared:** Writing – original draft; writing – review and editing. **Phool Iqbal:** Formal analysis; supervision; writing – original draft; writing – review and editing. **Ahmed Lutfe Mohamad Abdussalam:** Supervision; visualization.

## FUNDING INFORMATION

This research has been funded by Qatar National Library.

## CONFLICT OF INTEREST

The authors certify that they have no conflict of interest and no affiliations with or involvement in any organization or entity with any financial or nonfinancial interest in the subject matter or materials discussed in this manuscript.

## ETHICAL APPROVAL

The study is conducted ethically in accordance with the World Medical Association Declaration of Helsinki.

## CONSENT

Written informed consent was obtained from the patient to publish this report in accordance with the journal's patient consent policy.

## Data Availability

None.
